# Deep Insight of the Pathophysiology of Gestational Diabetes Mellitus

**DOI:** 10.3390/cells11172672

**Published:** 2022-08-28

**Authors:** Amarish Kumar Sharma, Sanjeev Singh, Himanshu Singh, Deviyani Mahajan, Prachetha Kolli, Gowtham Mandadapu, Bimlesh Kumar, Dharmendra Kumar, Sudarshan Kumar, Manoj Kumar Jena

**Affiliations:** 1 Department of Biotechnology, School of Bioengineering and Biosciences, Lovely Professional University, Phagwara 144411, Punjab, India; 2 Microgen Health Inc., 14225 Sullyfield Cir Suite E, Chantilly, VA 20151, USA; 3 Devansh Lab Werks, 234 Aquarius Drive, Homewood, AL 35209, USA; 4 School of Pharmaceutical Sciences, Lovely Professional University, Phagwara 144411, Punjab, India; 5 Animal Physiology and Reproduction Division, ICAR-Central Institute for Research on Buffaloes, Hisar 125001, Haryana, India; 6 Animal Biotechnology Centre, ICAR-National Dairy Research Institute, Karnal 132001, Haryana, India

**Keywords:** gestational diabetes mellitus, VEGF, pregnancy, pathophysiology, biomarker

## Abstract

Diabetes mellitus is a severe metabolic disorder, which consistently requires medical care and self-management to restrict complications, such as obesity, kidney damage and cardiovascular diseases. The subtype gestational diabetes mellitus (GDM) occurs during pregnancy, which severely affects both the mother and the growing foetus. Obesity, uncontrolled weight gain and advanced gestational age are the prominent risk factors for GDM, which lead to high rate of perinatal mortality and morbidity. In-depth understanding of the molecular mechanism involved in GDM will help researchers to design drugs for the optimal management of the condition without affecting the mother and foetus. This review article is focused on the molecular mechanism involved in the pathophysiology of GDM and the probable biomarkers, which can be helpful for the early diagnosis of the condition. The early diagnosis of the metabolic disorder, most preferably in first trimester of pregnancy, will lead to its effective long-term management, reducing foetal developmental complications and mortality along with safety measures for the mother.

## 1. Introduction

Pregnancy is one of the most complex physiological processes, which gives rise to a new progeny. Profound biochemical and physiological changes are observed throughout the pregnancy and a delicate balance is maintained among the biomolecules, such as pregnancy hormones and regulatory proteins, for a successful pregnancy. Some metabolic disorders are observed during pregnancy due to a lack of harmony among the regulatory molecules. Gestational diabetes mellitus (GDM) is a metabolic disorder where diabetes occurs during pregnancy, affecting 14% of total pregnancies globally, which corresponds to 18–20 million births annually [1]. Generally, a spontaneous hyperglycaemic condition develops during GDM and obesity, gradual overweight, high fat and low carbohydrate diet, previous diabetic history of parents and sedentary lifestyle are the major risk factors for the onset of this disease [2,3].

The American Diabetes Association (ADA) defines GDM as chronic glucose intolerance, which corresponds to the insulin resistivity of any degree, with the onset of pregnancy. Obesity is observed to have a long-term negative effect of GDM in females. GDM is an altered metabolic condition in which high blood sugar levels develop during pregnancy. It has no time-bound occurrence rate during the gestational period and can be trigger at any point in time. The main reason is the insufficient insulin secretion to meet up the demand of building blood glucose in the body during pregnancy. Elevated thirst level, frequent urination and dry mouth, along with continuous fatigue, are among the motr common symptoms of GDM. The pathophysiological effects of GDM, as per clinical studies, have been found to be temporary, but its aftereffects on maternal and foetal health are seen to be prominently negative [4].

As far as clinical observations are concerned, GDM is found to be temporary and is mostly resolved once the pregnancy period is over. However, there is a significant increase in metabolic complications, such as obesity, type 2 diabetes (T2D) mellitus and chronic cardiovascular disease. Currently, no medications are available to cure GDM completely except a healthy lifestyle (balanced diet and regular exercise), which also has a limited relief due to insulin resistance and further glucose intolerance [5,6].

The oral glucose tolerance test is a direct measure of the individual’s response to sugar intake and can be effectively used to diagnose GDM and T2D. An individual with GDM has fasting blood glucose level at approximately 105 mg/dL. The blood glucose level shifts from 190 mg/dL (1 h postprandial) to 165 mg/dL (2 h postprandial) and 145 mg/dL (3 h postprandial), respectively. Studies have revealed that during the first trimester of pregnancy the fasting plasma glucose (FPG) level remained more than 126 mg/dL (7.0 mmol/L), glycated haemoglobin level (HbA1C) at 6.5%, the random plasma glucose level at more than 200 mg/dL (11.1 mmol/L) and 2-h plasma glucose after a load of 75 g oral glucose remained over 200 mg/dL [7]. 

There has been an exponential rise in the GDM cases in the recent past due to an increase in the sedentary lifestyle, stress and the intake of a high calorific diet in the urban population, globally [8]. GDM also negatively affects the physiological and metabolic condition of the foetus developing inside the mother’s womb. There are controversies regarding optimal threshold criteria for screening the onset of GDM in late trimester, but there is clear evidence that early prediction and diagnosis results in successful treatment of the metabolic disorder [9]. Early prediction as early as first trimester would help medical professionals to identify, diagnose and eradicate the condition. Early prediction would allow preventive measures, such as diet control and other clinical interventions, to restrict GDM [10,11]. Therefore, in-depth research is required for the early prediction of GDM.

According to ADA, GDM is primarily diagnosed in the second or third trimester, but it is categorically undefined whether it is pre-existing type 1 diabetes or the T2D form. The exact threshold for the onset of GDM is not clear and various diagnostic criteria have been used to screen their presence in pregnant women [2]. As per recommendations of World Health Organization, the International Federation of Gynaecology and Obstetrics and ADA, the diagnosis criteria have been framed by the International Association of Diabetes and Pregnancy Study Group (IADPSG) [12]. As per diagnosis criteria recommended by IADPSG, all pregnant women should undergo a fasting plasma glucose (FPG) test. If the blood glucose reading is equal to or more than 92 mg/dL, it is an indication of GDM. Additionally, if the FPG level is less than 92 mg/dL two hours after the 75 g oral glucose tolerance test (OGTT) between 24 and 28 weeks of gestation, this is also indicative of the onset of GDM in pregnant women.

High blood glucose concentration in the first trimester may independently affect the onset of gestational diabetes, with the glucose cut-off at fasting lying in the range of 80–85 mg/dL [13]. The elevated levels of prediction biomarkers, such as the sex hormone-binding globulin (SHBG), C-Reactive Protein (CRP) and adiponectin, play a significant role in the diagnosis and pathogenesis of GDM. The sensitivity and specificity of SHBG and CRP are approximately 74% and 75%, respectively, in the 15th week of gestation in the pregnant individual, which can act as a potent biomarker for the early prediction of GDM [14].

## 2. Obesity, a Major Predisposing Factor for GDM

Obesity is a major risk factor for the onset of GDM, which is mainly due to the hyper production of pro-inflammatory cytokines from adipocytes, leading to chronic inflammation [15,16]. There is an active secretion of the tumour necrosis factor, pro-inflammatory interleukins, leptin, visfatin and adipokines as an outcome of chronic inflammation. Insulin secretion and its sensitivity, metabolic energy control and inflammation are controlled by adipokines [17,18,19]. Studies reveal the close relationship between the development of T2D, obesity and low-grade chronic inflammation [20]. The delicate interplay between the pro inflammatory and anti-inflammatory cytokines keeps the metabolic processes normal between mother and foetus during healthy pregnancy. Obese pregnant individuals release more pro-inflammatory cytokines, leading to metabolic imbalance, such as insulin resistance towards blood glucose in maternal as well as foetal blood [21]. The insulin resistance results in blood glucose build up and hyperglycaemia, which repeats the vicious cycle of obesity and other cardiovascular complications [22]. It is observed that the hyper-accumulation of proinflammatory cytokines promotes insulin resistance.

## 3. Insulin Resistance

**Figure 1:**
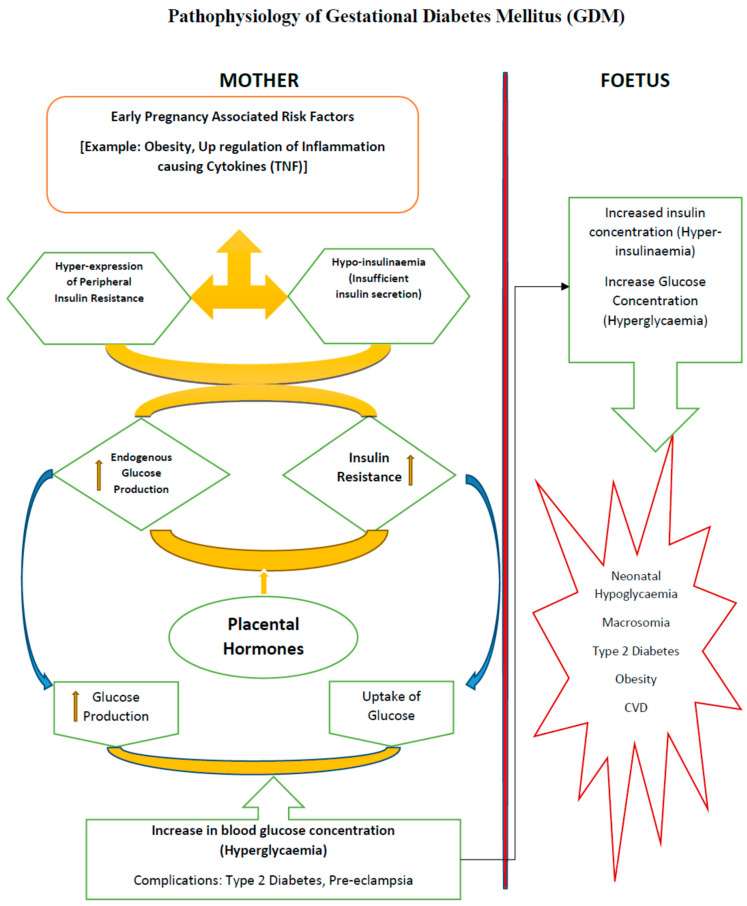
Physiological phenomena in Gestational Diabetes Mellitus (GDM). It has been evidenced experimentally that GDM affects women with dysfunctional metabolic systems before conception. During pregnancy, under hyperglycaemic overload and increased β-cell demand to compensate glucose uptake, endogenous insulin becomes unresponsive or undergoes increased insulin resistance, and is thereby unable to perform cellular glucose uptake through peripheral skeletal muscles and adipose tissue. The defects in the β-cell subsequently leads to insulin resistance in the maternal system, which leads to complications, such as hyperglycaemia and hyperinsulinemia, and may result in short term metabolic problems, such as foetal overgrowth or macrosomia, and long-term problems, including obesity and T2D.

The inability of insulin to perform cellular glucose uptake activity is termed insulin resistance, which may lead to onset of chronic conditions, such as obesity, cardiovascular complications and GDM in pregnant women (Figure 1). Insulin resistance may be due to excessive lipid or other metabolite accumulation, resulting in the activation of inflammatory signals ending up in endoplasmic reticular stress [23,24]. Different tissues respond differently to enhanced glucose flux, resulting in insulin resistance. In liver, enhanced sugar load promotes the partitioning of lipids away from mitochondrial oxidative pathway, thereby activating serine kinases and inactivating insulin signalling molecules. In muscles overloaded with fats, fatty acid oxidation is enhanced but Kreb’s downstream energy cycle is inhibited, resulting in the over-accumulation of unmetabolized lipid droplets in mitochondria, which ultimately leads to insulin resistance or insulin signalling impairment [25].

The insulin release in normal nonobese pregnant women is estimated through the hyperinsulinemic-euglycemic clamp study, and it was found that insulin sensitivity decreases by 56% through 34 to 36 weeks of gestation. It is important to note that approximately 39% of the total decrease in insulin sensitivity occurred in first 12–14 weeks of gestation. This decrease in insulin sensitivity induced a 3–3.5-fold increase in insulin release during the pregnancy [26]. Decreased insulin sensitivity towards blood glucose leads to over-storage in the body tissues, which further promotes obesity in women of reproductive age. Chronic obesity further paves the way for long term complications, such as cardiovascular disease, liver and kidney disorders [27]. 

Insulin insensitivity towards blood glucose is a major concern in GDM cases. TNF-alpha, which is produced by monocytes and macrophages, impairs the insulin sensitivity at high concentration, indirectly developing a hyperglycaemic condition, which leads to GDM in pregnant women. Experimental investigations have also proved that the impaired metabolism of blood glucose and increased oxidative cellular stress are associated with the early onset of GDM in pregnant women [28]. Studies have also revealed that high TNF-alpha concentrations in the blood negatively control or downregulate insulin sensitivity in pregnant women affected with GDM in the late 34th week [29]. The acute phase protein CRP is secreted during cell injury or microbial infection is found to be associated with obesity, and its high concentration in blood leads to insulin insensitivity towards blood glucose. A study revealed that there is a positive correlation between GDM with an elevated concentration of CRP and an individual’s BMI. This study concluded that there is a significant relationship between glucose intolerance or insulin resistance in the third trimester and a high CRP concentration in first trimester [30]. It has been observed that onset of GDM is due to the activation of inflammatory cytokines, such as IL-6 and TNF-alpha and the consistent downregulation of IL-4 and IL-10 [29,31].

Insulin signalling induces the translocation of the glucose transporter (GLUT) proteins, which further opens the plasma membrane for glucose uptake into the cell. The failure of the insulin signalling reduces the glucose absorption by the cell, leading to the onset of T2D, obesity and GDM in pregnant individuals. It is commonly observed that the rate of glucose absorption is drastically reduced by 54 percent in pregnant women affected with GDM compared to healthy women. Alterations in the downstream regulators of insulin signalling, mutations in insulin receptor substrate (IRS), endoplasmic reticulum stress during insulin processing and pro-inflammatory cytokines are the major reasons for the onset of GDM [32].

It is observed that the women who were normoglycaemic before pregnancy developed GDM in the late gestational period, which may be due to suppressed insulin sensitivity for blood glucose before conception. At an early gestation it was found that women affected with GDM maintained normal blood glucose levels, which may be due to the ability of the pancreas to stimulate β-cells to enhance insulin secretion. With the progression of pregnancy and the increased caloric intake for developing the embryo inside the mother’s womb, endogenous glucose production is increased by 30–35% and the insulin response to the presence of blood glucose decreases, which may be due to insulin resistance or β-cell dysfunction [24]. The dysfunctional pancreatic β-cells might exist before pregnancy, but its clinical manifestation is observed in late pregnancy, resulting in hyperglycaemia. In contrast to this, women diagnosed with GDM have been found to have a fasting hyperglycaemic condition, which might be induced due to suppressed endogenous glucose production before conception [33].

In healthy pregnant women, glucose uptake is initiated once insulin binds to its receptor embedded in the peripheral tissues, which phosphorylates its tyrosine kinase domain, resulting in the activation of the signalling cascade and the flux of GLUT molecules to the cellular membrane and the opening of the membrane gates for glucose uptake. The process of glucose uptake progresses until the glucose homeostasis is achieved, and thereafter the insulin ligand becomes detached from its receptor and the signalling process is switched off. It was found that the expression of the insulin receptor substrate-1 (IRS-1) in skeletal muscles decreases in pregnant females during last quarter of gestation as compared with non-pregnant healthy females, thereby lowering the insulin receptor-β (IR-β) phosphorylation, resulting in a 25–30% decreased glucose uptake [23,25].

## 4. Adipokines and Hormones in the Pathophysiology of GDM

Adiponectin plays a very crucial physiological role in the insulin sensitization for blood glucose. Moreover, it plays an anti-atherogenic and anti-inflammatory role in maintaining the immunological interplay between maternal and foetal blood across the placental barrier [34,35]. There is significant downregulation of adiponectin in hypertension, cardiovascular complications and obesity due to T2D and/or GDM [36,37]. Adiponectin can be an effective biomarker for the prediction of onset of GDM [38,39,40,41]. Further investigations are required for the early prediction of GDM as adiponectin level rise during the 25–28th weeks of pregnancy.

An adipocyte-derived hormone leptin under hypothalamic control plays a significant role in metabolic feedback regulation, which controls food intake. The malfunctioning of leptin can lead to irregular food intake and chronic obesity [42,43]. The overexpression of leptin promotes weight gain and obesity, which may force the pancreatic system to secrete more insulin in the blood, a condition termed hyperinsulinemia. The blood concentration of TNF-alpha and IL-6 increases under the hyperconcentration of leptin. Studies revealed that the leptin concentration is very high in obese pregnant females affected with GDM [44,45].

Visfatin, a visceral fat-derived adipokine, shows increased concentration in hyperglycaemia and obesity and is inconsistent in GDM [46,47]. Its expression is more prominent in first and second trimester of pregnancy and decreases gradually in the third trimester [48].

The protein SHBG has an inverse association with insulin. The normal concentration of SHBG found in females is in the range of 18 to 144 nmol/L. It has been observed that the condition of insulin resistance is very much eminent in pregnant individuals with low SHBG levels, which progressively leads to obesity and GDM. It has been found that low levels of SHBG in the 13th or 16th week may lead to the onset of GDM [49].

It has been investigated that as the pregnancy progresses, there is an upregulation of factors such as human placental lactogen (hPL), oestrogen, progesterone, cortisol and prolactin, respectively, which might decrease the peripheral insulin sensitivity [50]. Decreased pancreatic β-cell dysfunction, upregulated insulin expression and glucose-stimulated insulin secretion may result in hyperinsulinemia, leading to insulin resistivity for blood glucose. This unstable metabolic condition elevates the blood glucose and free fatty acid level, promoting GDM in pregnant females. In GDM-associated pregnancy, there is a significant increase in adipocyte fatty acid binding protein expression and decrease in PPAP-γ expression and chronic inflammation due to defective IRS-1 function and insulin receptor phosphorylation [51]. 

## 5. Foetal Derangements in GDM

Changes in the developing foetus vasculature may happen due to the changes in the feto-placental vessels in GDM-affected females. This leads to foetal derangements, hyperglycaemia and further metabolic complications. The hyperglycaemic condition in foetus induces higher insulin secretion, resulting in hyperinsulinemia in second trimester [52]. A GDM-affected woman generally delivers a baby of enlarged size, causing painful delivery and caesarean section. A condition defined as “Polyhydramnios”, which leads to the hyperaccumulation of amniotic fluid, which acts as a cushion and nurtures the foetus inside the mother’s womb, may act negatively and lead to premature labour or delivery complications. Premature birth, in or before the 37th week, and another chronic medical complication termed “Preeclampsia”, which primarily happens due to the onset of high blood pressure and the hyperconcentration of protein in urine, are common in females affected with GDM [53,54].

## 6. Pathophysiology and Pharmacological Interventions in GDM

**Figure 2:**
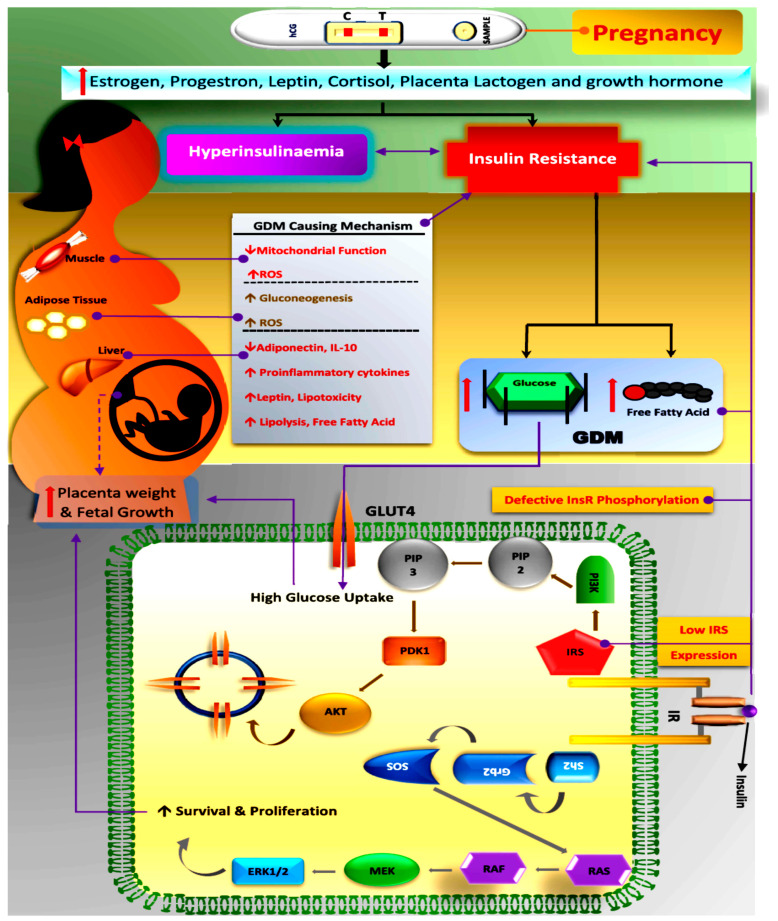
Schematic diagram showing the pathophysiology in the GDM-affected female, highlighting factors inducing hyperinsulinemia and insulin resistance. Glucose uptake via insulin signalling takes place through the auto-phosphorylation of the insulin receptor, thereby activating and recruiting GLUT molecules to open cell channels/gates for glucose uptake in skeletal muscles. Downstream effectors, such as IRS-1 and phosphatidyl inositol 3-kinase (PI3K), are major players that are activated, resulting in GLUT recruitment, which opens the membrane channels for glucose uptake intracellularly. GDM-affected pregnant females in late gestation exhibit regressed IRS1 expression, which minimizes tyrosine phosphorylation in a β subunit of the insulin receptor, which finally results in increased glucose tolerance or insulin resistance. The most visible complication of GDM is increased placental weight and an oversized foetus, which is due to high glucose uptake. (GDM: Gestational Diabetes Mellitus; ROS: Reactive Oxygen Species; IL: Interleukin; GLUT: Glucose Transporter Protein; PIP: Phosphatidylinositol phosphate; PI3K: Phosphoinositide 3-kinases; PDK1: Pyruvate Dehydrogenase Kinase; AKT: Protein Kinase B, also known as AKT; SOS: Son of Sevenless; Grb2: Growth Factor Receptor-Bound Protein 2; Sh2: Src Homology 2; RAS: Rat Sarcoma Virus; RAF: Rapidly Accelerated Fibrosarcoma; MEK: Mitogen-Activated Protein Kinases; ERK1/2: Extracellular Signal-Regulated Kinase 1/2; InsR: Insulin Receptor; IRS: Insulin Receptor Substate).

GDM is a silent glucose intolerance complication, which slowly creeps in pregnant females with varying severity. The onset of GDM is mostly recognised or diagnosed during the last trimester of gestation, leading to dual complications both in the mother and the developing foetus in the mother’s womb. Scientific studies have pointed to chronic insulin resistance and pancreatic β-cell dysfunction (Figure 2) as the main culprits for the onset of GDM, but the actual cause is still not understood and there is a wide scope for researchers to look into the precise biomarkers, which can detect its presence as early as possible during gestation [55]. There are hidden cellular events caused by a maternal genetic predisposition, coupled with the cellular micro-environment and feto-placental factors, which lead to GDM complications that are diagnosed in late gestation. Therefore, understanding the cellular pathophysiology and associated risk factors is very important for the effective screening and early intervention of GDM.

The sole function of pancreatic β cells is to sense blood glucose concentration and secrete insulin accordingly. The β-cell dysfunction is a condition of excessive insulin secretion in response to chronic blood glucose concentration and it has many manifestations, such as inappropriate pro-insulin synthesis, altered post-translational modifications, glucose intolerance and other unknown cellular machinery [56].

Recent studies have shown that genes, such as the potassium voltage-gated channel KQT-like 1 (Kcnq1) and Glucokinase (Gck), are susceptible to β-cell function and responsible to cause GDM in pregnant women [57].

Elevated hyperglycaemic blood load due to dysfunction in β-cell is exacerbated by insulin insensitivity towards blood glucose, which leads to an altered metabolic condition called glucotoxicity [58]. In vivo studies on rat models (Zucker fatty rats) suggest that β-cell number is an important parameter for homeostatic glucose control. In a study with Zucker fatty rats, 60% of the pancreatic system was excised through surgery and subsequently the β-cell mass was recovered to normal numbers, but the rat still developed severe hyperglycaemia. This study concludes that the sudden dramatic reduction in β-cell numbers may have overburdened the remaining β-cells, leading to glucotoxicity due to reduced insulin sensitivity or the depletion of insulin granules store [59].

An experimental study on Sprague Dawley rats, considered to be resistant to diabetic development, restricted their growth in utero via bilateral uterine ligation and a substantial loss (50%) of β cells was observed. This loss might be due to the epigenetic downregulation of Pdx1, a pancreatic transcription factor, which is essential for β-cell proliferation and differentiation in the embryonic stage [60]. The β-cell proliferation is also supported by the infusion of prolactin, as observed in a mouse knock-out model of the prolactin receptor [61]. Reduced β-cell hyperplasia and glucotoxicity are a few experimental interpretations, which prove that β-cell degeneration through apoptotic pathway may lead to the onset of GDM [32,62].

Foetal development is entirely dependent on maternal nutrient supply, mainly blood glucose. Hyperinsulinemic conditions are triggered when the growing foetus is overexposed to a hyperglycaemic environment in GDM-affected women. On the placental surface, endothelial lipase-mediated lipolysis induces the release of maternal lipoproteins, but only a small proportion of free fatty acids travels across the placental wall, where they build up a foetal free fatty acid pool, using excess blood glucose, which arises due to increased dietary intake during pregnancy. Adipogenesis is stimulated by foetal insulin and the resulting fat is stored in adipose tissues.

## 7. Vascular Endothelial Growth Factor—A Silent Player in GDM

Vascular Endothelial Growth Factor (VEGF) plays a significant role in placental angiogenesis. Recent studies have proved that abnormal angiogenesis could result in GDM due to altered blood glucose metabolism. It is also observed that there is incremental weight gain in the placenta of women with GDM as compared to a healthy woman [63]. Enhanced VEGF concentration induces placental hypervascularization and, in addition, the hyperglycaemic condition creates oxidative stress, resulting in the high sequestration of reactive oxygen species (ROS) and nitric oxide (NO), which play a critical role in feto-placental endothelial dysfunction, leading to metabolic complications during GDM [64,65,66]. It has been observed that in GDM-affected women, the A_2A_AR/NO/VEGF signalling pathway plays a significant role in the regulation of cell proliferation and migration. The synthesis of VEGF is enhanced through this pathway upon the interaction of adenosine or adenosine agonist with an adenosine receptor, thereby triggering serine 1177 and endothelial nitric oxide synthase (eNOS) phosphorylation, which results in the secretion of NO and nitrated tyrosine residues. It is predicted that cell proliferation and migration is amplified due to the enhanced expression of eNOS, NO formation and tyrosine nitration [67,68].

An in vitro human umbilical vein endothelial cell (HUVEC) study, carried out to observe the effect of a high blood glucose concentration on placental angiogenesis in the presence of fibroblast growth factor 2 (FGF2) and VEGF, revealed that cell proliferation is reduced under the hyperglycaemic condition as compared to the healthy state [69]. A similar study also showed that cell proliferation was suppressed under FGF2 stimulation in HUVEC in GDM pregnancy models as compared to control, under high blood glucose (25 mM) levels [70]. One more interesting finding showed that the rate of cell proliferation was unaffected under VEGF-stimulated HUVEC in hyperglycaemic GDM cell culture models [71]. A further study revealed that cell proliferation under VEGF stimulation was unaffected in the hyperglycaemic condition, and there was a sharp rise in the VEGF mRNA concentration in the HUVEC diabetic model [72].

The transcriptional deregulation of genes controlling cellular movement, adhesion and migration was observed under the hyperglycaemic condition in HUVEC, which minimizes the migratory potential of diabetic HUVEC, but no effect was observed in normal HUVEC. The above investigation proves a loss in basal migratory activity under the hyperglycaemic condition, particularly in GDM. Moreover, the rate of placental angiogenesis under high blood glucose concentrations is highly compromised and a reduction in basal migratory capacity could be one factor contributing to this outcome [73,74].

The mitogen-activated protein Kinase 1/2 (MEK1/2), an extracellular signal-regulated Kinase 1/2 (ERK1/2), also known as the Ras-Raf-MEK-ERK pathway, is a signalling pathway which communicates cellular signals from the cell surface to the nuclear DNA and plays a very important role in cell proliferation, differentiation and survival. It was revealed that MEK1/2/-ERK1/2 is involved in VEGF-induced feto-placental endothelial cell proliferation and differentiation and GDM inhibits this signalling pathway with significant reduction in cell multiplication and localization under FGF2 activation [75].

Notch signalling plays a vital role in angiogenesis via VEGF regulation. Reduction in Flt-1 expression with no change in VEGF or kinase insert domain receptor (KDR) expression was observed in gestational diabetes. No significant change in cell proliferation was observed but migration was higher as compared to normal pregnancies. The activation of KDR, promoting the overstimulation of the migration of endothelial cells, leads to the hypervascularization of placenta in GDM-affected individuals. Capillary branching is more prominent in GDM-affected individuals and the incidence of chorioangioma (a metabolic unstable condition with an abnormal increase in the number of vascular channels in the villi of the placenta) becomes significantly higher [76]. Simultaneously increased villous immaturity and a sharp rise in placental angiogenesis under the hyperglycaemic condition could serve as early physiological predictions for the onset of GDM in pregnant women [77].

The placental studies on the expression of VEGF-A and its corresponding receptor VEGFR-2 (KDR) in GDM revealed that there is reduced expression of both the VEGF-A and VEGFR-2 in GDM pregnancies as compared to those of normal pregnancies [78]. This finding indicates that GDM affects the pathophysiological function of placenta. So far, it is not clear about the factor which might control the placental levels of VEGF and its receptors in GDM-affected individuals [79,80]. Another study revealed that VEGF might be the leading factor for the enhanced vascularization of placental tissues in individuals with GDM, showing a higher expression of KDR and a reduced expression of Flt1 protein [81].

Single nucleotide polymorphisms (SNPs) are the biomarkers of genetic variation among individuals. Studies on SNP help understand the pathophysiology of disease, such as T2D and GDM at the genetic level [82]. SNPs, such as rs2146323 and rs3025039, which prominently occur in the VEGF gene, could be the major factor for the onset of GDM in pregnant women. The rs3025039 SNP present in the VEGF gene is found to be main culprit in the pathogenesis of the metabolic disorder [83]. Gene polymorphism in the VEGF gene could induce abnormality in its expression and may lead to the development of metabolic disorders. It has been proven in previous studies that the hyperconcentration of VEGF in serum is a probable biomarker for the onset of GDM and diabetic polyneuropathy [8,9,49]. Gene polymorphic form rs3025039 within the VEGF gene may be a crucial factor affecting its serum concentration. The rs3025039 form is located in the 3′-UTR region, where SNP affects the genetic stability via microRNA and mRNA interaction [84,85]. In another study it is revealed that the VEGF gene’s polymorphic form, rs3025039, affects the cellular pathophysiology of many diseases, including GDM, by downregulating the VEGF transcriptional level and interacting with other SNPs. Linkage disequilibrium among rs2010963, rs833069, rs2146323 and rs3025010 was found, which may lead to the onset of metabolic disorders. Current in silico research investigations concluded that BMI, HOMA-IR, rs2146323, rs3025039, CT + TT genotype and VEGF activity are independent risk factors and screening parameters for the diagnosis of GDM, while HOMA-beta is an independent protective factor of GDM and an index of insulin secretory function. It is used to predict diabetes development [86,87]. It can be summarized from the above findings that VEGF gene loci play a significant role in the pathogenesis of GDM and more research work is required to screen multiple loci, which have role in the pathophysiology of this metabolic disorder.

There is clinical evidence of the decreased concentration of VEGF in cord plasma isolated from GDM-affected women [88]. Hypoxia or oxidative stress upregulates VEGF expression levels, as they are oxygen dependent, thereby upregulating the process of placental and foetal angiogenesis.

## 8. Predictive Biomarkers for the Early Diagnosis of GDM

A hyperglycaemic condition in the mother could be the major factor which induces feto-placental endothelial dysfunction, where the MEK1/2-ERK1-2 signalling pathway is highly compromised. Under the hyperglycaemic condition, the inhibition of the MEK1/2-ERK1-2 pathway suppresses cell proliferation under fibroblast growth factor (FGF) stimulation. It is revealed that high blood glucose concentration and gestational diabetes in obese models and inhibits the signalling of FGF2 but not of VEGF, which is significantly responsible for ex vivo angiogenesis [89].

The oral glucose tolerance test (OGTT) is the gold standard for the pre-screening of GDM but carries low reproducibility and is time consuming. Hence, it becomes inevitable to look for predictive biomarkers for the early diagnosis of GDM. A list of biomarkers compiled in a review article authored by Huhn et al. (2018) provides future directions for further research work on biomarker discovery and their adoption in clinical diagnosis for the early detection of GDM [90].

The hyperglycaemic condition during pregnancy affects foetal development significantly. Pathological screening tests for blood glucose is performed in late second or early third trimester to diagnose GDM. The early prediction of GDM could serve significantly to restrict future complications at a very early stage. Prenatal screening in the first trimester of the gestation period to detect any defects of foetal aneuploidy is widely accepted obstetric practice [91,92]. Pregnancy-associated plasma protein (PAPP-A) and free beta-human chorionic gonadotropin hormones from the maternal serum, which are generally secreted from placenta, are the potent biomarkers for the screening of the foetal aneuploidy [93,94]. Moreover, free beta-hCG plays a crucial role in placental development and can serve as an essential early biomarker in the identification of GDM. 

Meta-analysis results are considered viable and reproducible only when they are subjected to large and diverse populations. Early prediction biomarkers can be used for the diagnosis and screening of both mother and child in regard to any potential metabolic complications [95].

Myostatin and amyokine, which are released from myocytes and inhibit myogenesis, is found to play a crucial role in glucose uptake in muscle and fat cells, respectively [96,97]. A study revealed that under high calorific meal (high fat), myostatin inhibition improves insulin sensitivity, which directly promotes blood glucose absorption facilitated through GLUT protein, a glucose transporter protein [98]. Myostatin is significantly expressed in the human placenta and plays a major role in glucose homeostasis, suggesting that it can serve as potent biomarker for the diagnosis of GDM in the early trimester [99].

## 9. Role of microRNA in GDM

The physiological role of microRNAs (miRNAs) is highly diverse, and they play a crucial function in physiological processes, such as the regulation of growth and development, differentiation, cellular homeostasis and epigenetic regulation. They have a role in pancreatic development as well as the glucose-induced secretion of insulin. 

The medical diagnosis of GDM is tentatively performed around the second and third trimester, which might be too late to overcome certain maternal or foetal complications. Present trends in GDM investigations are gaining impetus where novel early gestation cellular markers are looked into with serious concern as they may cause serious and irreversible maternal or foetal complications post-childbirth. The miRNAs can be used as potential markers for the early screening of GDM [100]. A study revealed that the changes in the maternal metabolic processes might be directly linked with variations in the placental miRNA expression. The role of miR-98 is found to be significant in the initial stage of pregnancy. An in vitro study on JEG-3 cells has proven the critical role of Trpc3 in controlling insulin-mediated glucose uptake through regulating the transcription factor Mecp2. Moreover, miR-98 is also involved in the insulin-mediated glucose homeostasis in GDM-affected woman [101].

The study by Zhao et al. (2014) has shown that the upregulation of miR-518d leads to the repression of peroxisome proliferator-activated receptor-α (PPARα), a critical nuclear hormone receptor controlling glucose uptake in mammalian tissues. PPARα also plays a significant role in metabolic conditions, such as inflammation, oxidative stress and insulin signalling. Glucose intolerance in GDM-affected woman may be due to the downregulation of PPARα and Retinoid X receptors (RXR). Furthermore, GLUT protein, which plays a crucial role in opening the glucose channels in the plasma membrane during insulin-mediated glucose uptake, is inhibited under the upregulation of miR-222, leading to insulin resistance and further T2D or GDM complications [102].

The role of miRNA in pancreatic β-cell dysfunction has been critical, but its involvement in the pathogenesis has not been fully understood. The miR-138p is overexpressed during GDM, which inhibits the proliferative and migration ability of mammalian trophoblast cells (HTR-8/S Vneo) by targeting the miR-138p specific target TBL1X, which then activates the wnt-beta-catenin pathway, a key player in controlling cellular proliferation and differentiation [103].

The miR-5P is overexpressed in GDM and it targets many genes during fatty acid metabolism. The miR-20a-5p and miR-222-3P are repressed in GDM as compared to the control group, whereas the miR-7-5P is overexpressed in GDM [104].

Table 1 shows the list of miRNA that are involved in the pathophysiology of the GDM condition.

## 10. In Vitro and In Vivo Models for GDM Study 

In recent years, there has been significant progress in the development of in vitro and in vivo experimental models to investigate the pathophysiology of T2D and GDM. There are certain in vitro cultured cell models derived from mammalian cells which can help us understand the essential markers connected to maternal or foetal complications during pregnancy [117]. 

Cell lines, such as BRIN-BD11, are specifically a clonal insulin-secreting mammalian cell line under glucogenic stimulation and works as an effective pharmacological modulator. This cell line is widely used in drug target research and functionally mimics the pancreatic β-cells. INS-1 is another mammalian cell line obtained from rat insulinoma. It is a stable cell line with high proliferative capability but is less sensitive to glucose as compared to that of primary islets cells. Similarly, immortal continuous cell lines, such as PANC-1, are robust cell lines, expressing a wide range of genes and proteins connected to insulin production [118,119]. A choriocarcinoma placental cell line was used in many previous studies to profile the effect of glucose uptake and was used prominently in GDM-related studies [120]. The stability and ease of handling these transformed cell lines in terms of regular passaging and maintenance is much better as opposed to primary trophoblast cells, which are directly isolated from placental tissue. The biggest challenge when carrying out work on primary placental trophoblast cells is their low yield, cellular viability and limited life span [121,122]. Placental cell lines, such as BeWo, JEG and JAR cells have been used as cellular models to examine the myostatin mechanistic effect on glucose uptake. BeWo cells are highly accepted cell lines for human placental transport studies [123]. These cells facilitate glucose uptake in placental system through GLUT-1, -3 and -4 transporters [96,99]. Sw.71, the immortalized human trophoblast cells—hTERT, is derived from seven week (first trimester) placental tissue from a healthy pregnant woman and is immortalized by virally infecting it with hTERT. These cell lines are well characterized to mimic the physiological properties of extra-villous trophoblast cells [101,102,103]. The Sw.71 cell line is used as a potent tool to study trophoblast research and can help in the screening of early prediction biomarkers of GDM in the first trimester.

Surgically-induced in vivo rodent models are widely used animal GDM models and these models exhibit partial diabetic phenotypic expressions [124]. Dietary alteration is another way to generate a diabetic model in pregnant mice. Several approaches, such as continuous glucose infusion or high dietary fat, can induce obesity, which is a major marker for the onset of GDM in pregnant mice. High fat, high fructose diets develop T2D and GDM and are associated with other metabolic complications, such as hepatic fibrosis, oxidative stress and inflammation [125]. For placental studies, specifically in the case of GDM, the administration of STZ in pregnant mice is the main strategy to develop an in vivo diabetic mellitus model. Developing mice models following the above strategy not only uncovers structural and functional abnormalities but also abnormal genetic expressions under a particular metabolic condition [125]. In vivo models, such as CIMs and BB rats, are widely used to evaluate the intra-uterine environment impact on developing foetuses. The null mutant mouse model, Socs2, is found to be one of the more effective models for GDM associated with aging, as aged pregnant mice models show lower birth and high mortality rates and the chances of developing foetuses to be affected with macrosomia is very high [126]. Table 2 describes the in vivo and in vitro models used for studies related to GDM pathophysiology.

## 11. Future Perspectives

The early prediction of GDM, as early as the first trimester, would allow medical professionals to initiate prophylactic therapies and clinical interventions in high-risk populations to facilitate early diagnosis and complete treatment. Future research work needs to focus on the development of a sensitive and accurate strategy to predict, diagnose and treat the affected individuals suffering from GDM, and research on biomarker discovery can play the vital role. The treatment strategy should be adopted for the safeguarding of both mother and foetus. Moreover, targeted drug delivery to the placenta can promote disease management with minimal side effects. The early management of GDM will prevent the probable abnormality in foetal development at an early stage and can aid in taking precautionary steps to prevent any future chronic ailments, such as obesity, T2D or cardiovascular complications [133]. The development of efficient study models for GDM during the first trimester of pregnancy would help the understanding of the pathophysiology of the metabolic disorder in further molecular detail [95]. Future research work should be directed towards preventive strategies to control metabolic disorders, as we have at present with many infectious diseases.

## 12. Conclusions

This comprehensive review on GDM reveals the seriousness of this metabolic disorder, and the urgent attention of research groups is required for the present scenario. The early diagnosis strategy of GDM is still under study and in-depth research work is required to unravel the regulatory molecules promoting the disorder and to identify potential therapeutic targets for treatment. A meta-analysis study has been conducted for first trimester gestation period with inconsistent results. Further studies will help manage the condition more appropriately to safeguard the health of the mother and foetus.

## Figures and Tables

**Table 1:** List of miRNAs involved in the pathophysiology of GDM.

miRNA Isoform	Functional Role	References
miR-222	The downregulated miR-222 and upregulated CXCR4 promotes insulin sensitivity and inhibits apoptosis in GDM.	Shi et al., 2014 [105]
miR-98	Upregulation of miR-98 in the placental tissues of human GDM is linked to the global DNA methylation via targeting MECP2.	Cao et al., 2016 [106]
miR-518d	Increased expression of miR-518d is correlated with decreased protein expression of peroxisome proliferator-activated receptor-α (PPARα), a nuclear hormone receptor controlling glucose homeostasis.	Zhao et al., 2021 [100]
miR-340	Insulin increased and glucose reduced miR-340 expression in GDM-affected women.	Stirm et al., 2018 [107]
miR-130b	This miR reflects the degree of obesity and serves as a potential biomarker for the diagnosis of GDM.	Wang et al., 2013 [108]
miR-148a	Regulates AMPKα1 activity (AMPK activity is significantly reduced in adipose tissue and the skeletal muscle of GDM-affected women).	Tryggestad et al., 2016 [109]
miR-144	Upregulated in GDM-affected pregnant women.	Collares et al., 2013 [110]; Juchnicka et al., 2022 [111]
miR-517-5p	Expressed specifically in placenta; potential role in GDM pathogenesis.	Wander et al., 2017 [112]
miR-21-5p	Placental miR-21-5p expression inhibits cell growth and infiltration by upregulating PPAR-α. It is downregulated in GDM, thereby affecting the placental function.	Guan et al., 2020 [113]
miR-146b-5p	Has a pivotal role in pregnancy and pregnancy-related complications; associated only with GDM-affected patients carrying male foetuses	Zhu et al., 2018 [114]
miR-210-3p	Impairs pancreatic β-cell function by targeting Dtx1 in GDM	Cao et al., 2022 [115]
miR-222-3p	Upregulated expression of miR-222-3p is a potential circulating biomarker for the pathogenesis of GDM	Sadeghzadeh et al., 2020 [116]

**Table 2:** In vivo and in vitro models relevant to GDM study.

Experimental Model	Species	Model Type	Experimental Features	References
Streptozotocin-Induced Diabetes Model.	Rat	In vivo	To study pathophysiology of GDM	Damasceno et al., 2014 [127]
C57BL/6	Mouse	In vivo	Pregestational DM defined by non-fasting BG > 13.3 mmol/L	Chang et al., 2005 [128]
db/db	Mouse	In vivo	Obese, insulin resistant model with elevated glucose level; target gene is ObR gene.	Yamashita et al., 2001 [129]
PrlR^±^	Mouse	In vivo	Target gene is PrlR gene. Decreased β-cells, decreased tolerance to glucose; repressed insulin secretion; impaired glucose homeostasis.	Nteeba et al., 2019 [130]
HTR2b^−/−^	Mouse	In vivo	Target Gene is 5-HTR2b; decreased β-cell proliferation; high glucose intolerance.	Baeyens et al., 2016 [131]
CD1	Mouse	In vivo	Pregestational DM defined by non-fasting BG > 17.0 mmol/L	Ge et al., 2014 [132]
Murine β-cell lines	Rat/Mouse	In vitro	Easy to culture; good option to test drugs and study cell physiology	Lilao-Garzón et al., 2021 [117]
Human pancreatic islets	Human	In vitro	Maintain the islet structure and all cell types; used to study the biology of the human pancreas.	Lilao-Garzón et al., 2021 [117]

## Data Availability

Not applicable.
